# Overexpression of microRNA-423-3p indicates poor prognosis and promotes cell proliferation, migration, and invasion of lung cancer

**DOI:** 10.1186/s13000-019-0831-3

**Published:** 2019-06-04

**Authors:** Rukun Wang, Gaofeng Li, Guoyan Zhuang, Shuying Sun, Zhihui Song

**Affiliations:** 1 Department of Thoracic Surgery, Weifang Cancer Hospital, Weifang, 261041 Shandong China; 2 Department of Thyroid and Breast Surgery, Weifang Cancer Hospital, Weifang, 261041 Shandong China; 3 Department of Outpatient, Weifang Cancer Hospital, Weifang, 261041 Shandong China; 4 Department of Nursing, Weifang Cancer Hospital, Weifang, 261041 Shandong China

**Keywords:** Lung cancer, microRNA-423-3p, Prognosis, Proliferation, Migration, Invasion

## Abstract

**Background:**

Lung cancer is one of the common malignant tumors worldwide with high incidence and mortality. MicroRNA-423-3p (miR-423-3p) acts as an oncogene in several types of cancers. The aim of this study is to reveal the clinical significance and biological function of miR-423-3p in lung cancer.

**Methods:**

The expression of miR-423-3p was detected in lung cancer specimens by reverse transcription-quantitative polymerase chain reaction (qRT-PCR) assay. Kaplan-Meier survival and Cox regression analyses were used to investigate the prognostic significance of miR-423-3p in lung cancer. CCK-8 and Transwell assays were used to determine the functional role of miR-423-3p in lung cancer.

**Results:**

We observed that miR-423-3p was significantly upregulated in lung cancer tissues and cell lines. Overexpression of miR-423-3p was significantly associated with lymph node metastasis, TNM stage, and poor prognosis. Multivariate Cox regression analysis results showed that miR-423-3p was an independent prognostic indicator for lung cancer patients. Results of functional analyses revealed that overexpression of miR-423-3p promoted cell proliferation, migration, and invasion in lung cancer cells.

**Conclusions:**

These results indicated that miR-423-3p acts as an oncogene and promotes cell proliferation migration, and invasion of lung cancer. And miR-423-3p may serve as a potential prognostic biomarker and therapeutic target for the treatment of lung cancer.

## Introduction

Lung cancer is a leading cause of cancer-related deaths worldwide with the highest incidence and mortality [1]. The most effective treatment for patients at an early stage is surgical resection, but the relapse rate after surgery is high. In addition, numbers of patients are diagnosed at an advanced stage and the overall survival rate is poor [2, 3]. Lung cancer is also a severely healthy burden in China, the incidence and mortality rate is still increasing with the accelerated progress of China’s industrial development and changes in lifestyle [4–6]. Because of the poor survival of lung cancer patients in advanced stages, it is important to identify novel effective prognostic biomarkers and therapeutic strategies to improve the treatment of lung cancer.

MicroRNAs (miRNAs or miRs) are a class of small non-coding RNAs, approximately 22 nucleotides in length that negatively regulate gene expression at the post-transcriptional level through binding to the 3′- Untranslated Region(UTR) of target mRNAs [7, 8]. Emerging evidence indicated that miRNAs play critical roles in the development and progression of various kinds of diseases, including cancers [9–11]. Numerous studies have indicated that the aberrant expression of miRNAs is involved in various biological processes, including development, proliferation, migration, invasion, and apoptosis [12–14]. Recent studies demonstrated that miRNAs such as miR-210, miR-577, and miR-141-3p are abnormal expressed in lung cancer pathogenesis [15–17]. A recent study by Zhu et al. showed that ten miRNAs including miR-423-3p are increased in serum of lung cancer patients [18]. However, the expression pattern of miR-423-3p in lung cancer tissues and biological role of miR-423-3p in lung cancer is still unclear.

In the present study, we aimed to investigate the expression and functional role of miR-423-3p in lung cancer and highlight its potential as a biomarker for lung cancer.

## Materials and methods

### Patients and tissue specimens

A total of 126 patients with lung cancer were included between January 2011 and December 2012 at Weifang Cancer Hospital in this study. The paired lung cancer tissue and matched adjacent normal tissue specimens (all were confirmed by at least two pathologists) were collected during surgery and snap frozen and stored at − 80 °C until RNA extraction. All the patients did not receive any treatment before surgery. 5-year follow-up information was enrolled to collect survival status by telephone. The clinicopathological characters of lung cancer patients were collected and listed in Table 1. All participants signed written informed consent in this study. The research was approved by the Ethics Committee of Weifang Cancer Hospital.

**Table 1:** Correlation between miR-423-3p expression levels and clinical features in lung cancer patients

Parameters	Cases No. (*n* = 126)	miR-423-3p expression		*P*
		Low (*n* = 61)	High (*n* = 65)	
Age				
< 60	66	29	37	0.292
≥ 60	60	32	28	
Gender				
Male	75	37	38	0.802
Female	51	24	27	
Tumor size				
< 4 cm	69	37	32	0.198
≥ 4 cm	57	24	33	
Differentiation				
Well + Moderate	77	39	38	0.529
Poor	49	22	27	
Lymph node metastasis				
Negative	67	39	28	0.019
Positive	59	22	37	
TNM stage				
I - II	73	44	29	0.002
III - IV	53	17	36	

### Cell lines and transfection

Four lung cancer cell lines (A549, H1299, HCC827, A427) and one human lung epithelial BEAS-2B cell line were purchased from Shanghai Cell Bank of the Chinese Academy of Sciences. All cells were cultured in RPMI 1640 medium (Gibco/Life Technologies, Grand Island, NY) supplemented with 10% fetal bovine serum (FBS) at 37 °C in a humidified atmosphere with 5% CO_2_. miR-423-3p mimic, mimic negative control (NC), miR-423-3p inhibitor, inhibitor NC (RiboBio, Guangzhou, China) were used for the overexpression and knockdown of miR-423-3p. Lung cancer cells were seeded onto a 6-well plate at 40–50% confluence before indicated transfection. Transfection of cells was conducted using Lipofectamine 2000 Reagent (Invitrogen; Thermo Fisher Scientific, Inc.) according to the manufacturer’s instructions. Untreated cells were used as control.

### RNA extraction and qRT-PCR analysis

Total RNA was extracted from tissues or cells by using TRIzol reagent (Thermo Fisher Scientific, Waltham, MA, USA) following the manufacturer’s protocol. RNA concentrations were determined using the NanoDrop. Purified total RNA was reverse transcribed to complementary DNA (cDNA) using a High Capacity cDNA Reverse Transcription Kit (Applied Biosystems, Foster City, CA). Expression of miR-423-3p was measured by an SYBR Green I Real-Time PCR Kit (GenePharma, Shanghai, China) on an Applied Biosystems 7900 Real-Time PCR system (Applied Biosystems, Foster City, CA). Relative miR-423-3p expression was normalized to the expression of U6 and was quantified with the 2^−ΔΔCt^ methods.

### Cell proliferation assay

CCK-8 assay (Dojindo Molecular Technologies, Inc., Kumamoto, Japan) was used to assess the cell proliferation capacity following the manufacturer’s protocol. Transfected cells were seeded into 96-well culture plates (1 × 10^4^ cells/well) at 37 °C in a humidified atmosphere with 5% CO_2_. Then CCK-8 reagents were added into cells at 0, 24, 48, and 72 h. After incubated for 1 h, the absorbance was measured using a microplate reader (Thermo Fisher Scientific) at 450 nm wavelength. The assay was repeated at least three times at each time point.

### Cell migration and invasion assay

Transwell chambers (Multiskan MK3, Thermo, Waltham, MA, USA) with a pore size of 8 μm were used for cell migration and invasion assays. For the migration assay, no Matrigel was used in the top chamber, while for the invasion assay, Matrigel (BD Biosciences, Franklin Lakes, NJ, USA) was pre-coated in the top chamber. After 24 h of transfection, cells (5 × 10^4^ cells/well) were seeded into the upper chamber with serum-free growth medium, and the lower chamber was filled with 500 μl medium with 10% FBS as a chemoattractant. Cells that were adhered to the lower membrane were fixed in 3.7% formaldehyde for 5 min and stained with 0.1% crystal violet for 15 min. Stained cells were counted under a microscope.

### Statistical analysis

SPSS 20.0 software (SPSS, Inc., Chicago, IL, USA) and GraphPad Prism 5.0 software (GraphPad Software, Inc., Chicago, USA) were used for statistical analysis. Data are presented as mean ± standard deviation (SD). χ^2^ test, Student’s t-test or one-way analysis of variance (ANOVA) followed by Tukey’s *post-hoc* test was applied to evaluate statistical differences. Kaplan-Meier analysis and Cox regression analysis were used to determine the prognostic significance of miR-423-3p. Differences were considered statistically significant when *P* < 0.05.

## Results

### Expression of miR-423-3p in lung cancer tissues and cell lines

**Fig. 1:**
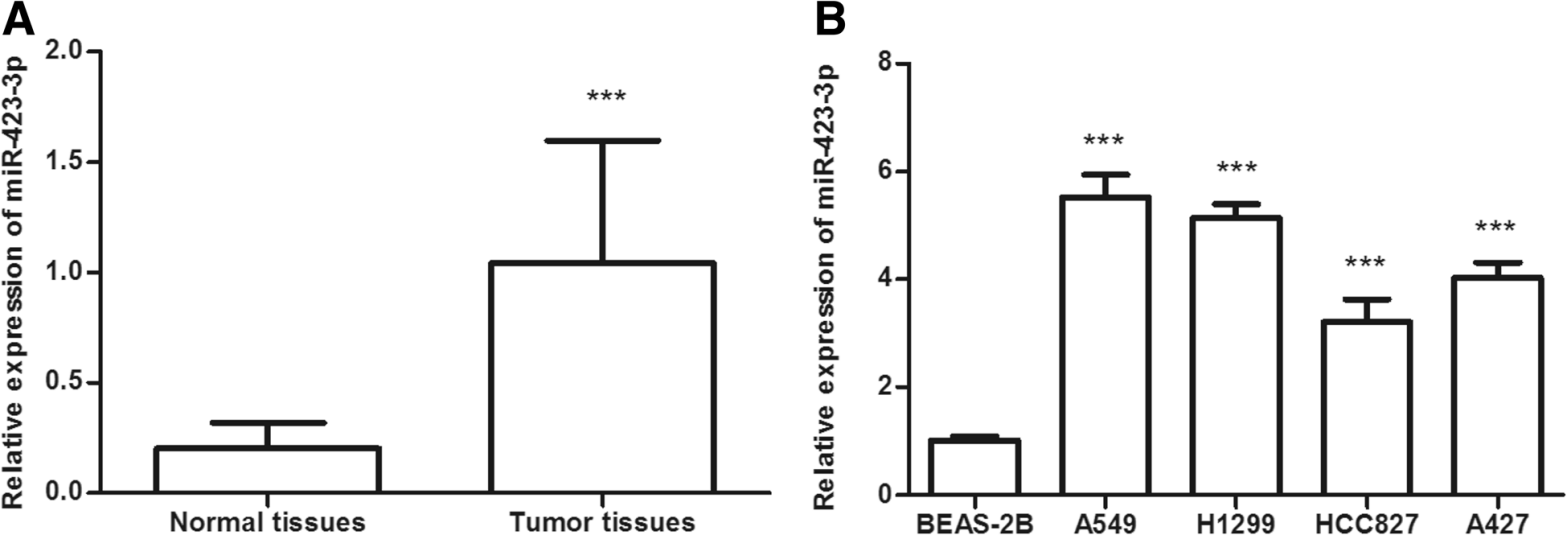
Expression of miR-423-3p in lung cancer tissues and cell lines. **a** Expression of miR-423-3p was upregulated in lung cancer tissues compared with normal tissues. (***P < 0.001). **b** Expression of miR-423-3p was increased in lung cancer cell lines (A549, H1299, HCC827, and A427) compared with normal cell line BEAS-2B. (***P < 0.001)

At first, the expression of miR-423-3p was measured in lung cancer tissues and adjacent normal tissues by qRT-PCR analysis. The results revealed that the expression of miR-423-3p was significantly increased in the lung cancer tissues compared with adjacent normal tissues (*P* < 0.001, Fig. 1a). Then, we assessed the expression of miR-423-3p in four different lung cancer cell lines and one normal cell line. As shown in Fig. 1b, the expression of miR-423-3p was significantly upregulated in all the lung cancer cell lines compared to the normal cell line (*P* < 0.001). Among the lung cancer cell lines, the highest expression levels of miR-423-3p were observed in A549 cell line, followed by the H1299 cell line. As the two cell lines have a relatively higher expression of miR-423-3p, both of them were used for subsequent experiments.

### The expression of miR-423-3p was correlated with clinicopathological features of lung cancer patients

In addition, the association between miR-423-3p expression and clinical features of lung cancer patients was analyzed. All the patients were divided into a low expression of the miR-423-3p group and high expression group according to the mean miR-423-3p expression value used as a cutoff. Using the χ^2^ test, we found upregulation of miR-423-3p was significantly associated with lymph node metastasis (*P* = 0.019) and tumor, node, and metastasis (TNM) stage (*P* = 0.002) (Table 1). However, the expression of miR-423-3p has no significant association with other features, such as age, gender, tumor size, and tumor differentiation (all *P* > 0.05, Table 1).

### miR-423-3p was correlated with poor prognosis in lung cancer patients

**Fig. 2:**
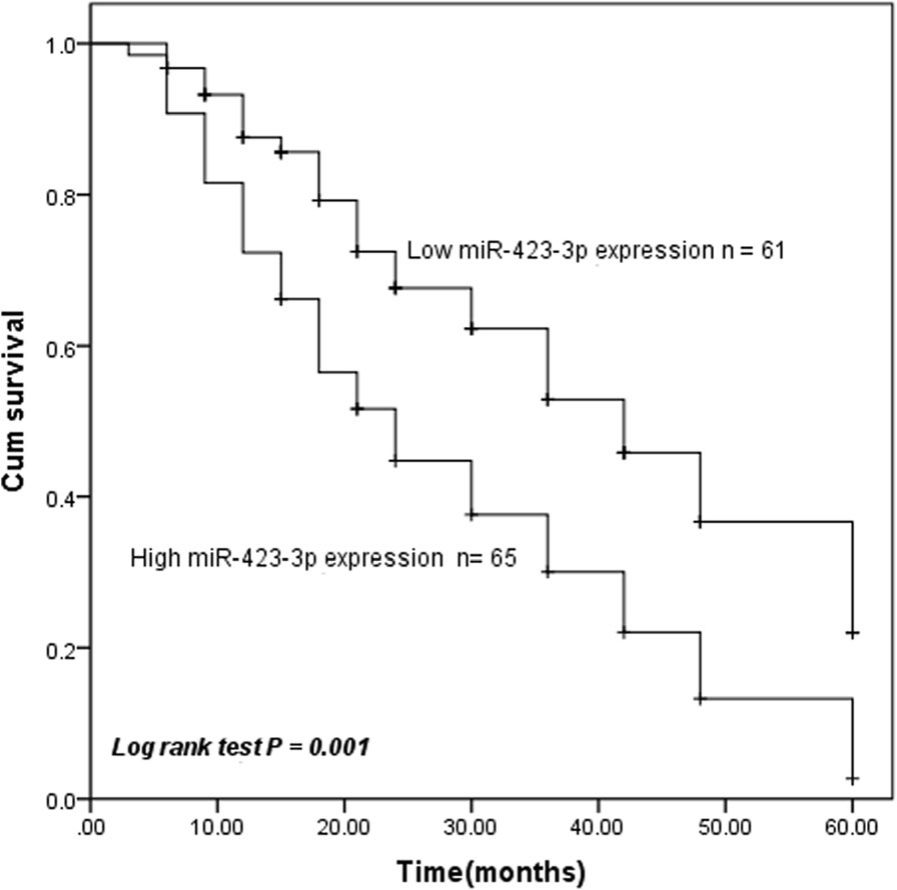
Upregulation of miR-423-3p correlated with poor prognosis of lung cancer patients according to the result of the Kaplan-Meier method

We then used Kaplan-Meier analysis to evaluate the prognostic significance of miR-423-3p in lung cancer according to the miR-423-3p expression and overall survival information of lung cancer patients. The results showed that the 5-year overall survival rates of lung cancer patients with high expression of miR-423-3p were shorter than those with low expression of miR-423-3p (log rank test *P* = 0.001, Fig. 2). Next, multivariate Cox’s hazard regression analysis results indicated miR-423-3p expression level (HR = 2.217, 95%CI = 1.344–3.659, *P* = 0.002) and TNM stage (HR = 0.585, 95%CI = 0.357–0.959, *P* = 0.034) are independent prognostic factors for 5-year overall survival in lung cancer patients (Table 2).

**Table 2:** Multivariate Cox analysis of clinical parameters in relation to overall survival

Characteristics	Multivariate analysis		
	HR	95% CI	*P*
miR-423-3p	2.217	1.344–3.659	0.002
Age	0.981	0.619–1.553	0.933
Gender	0.945	0.597–1.494	0.808
Tumor size (cm)	1.445	0.918–2.273	0.111
Differentiation	1.032	0.637–1.671	0.898
Lymph node metastasis	1.158	0.726–1.847	0.539
TNM stage	0.585	0.357–0.959	0.034

### miR-423-3p promoted lung cancer cell proliferation, migration, and invasion

**Fig. 3:**
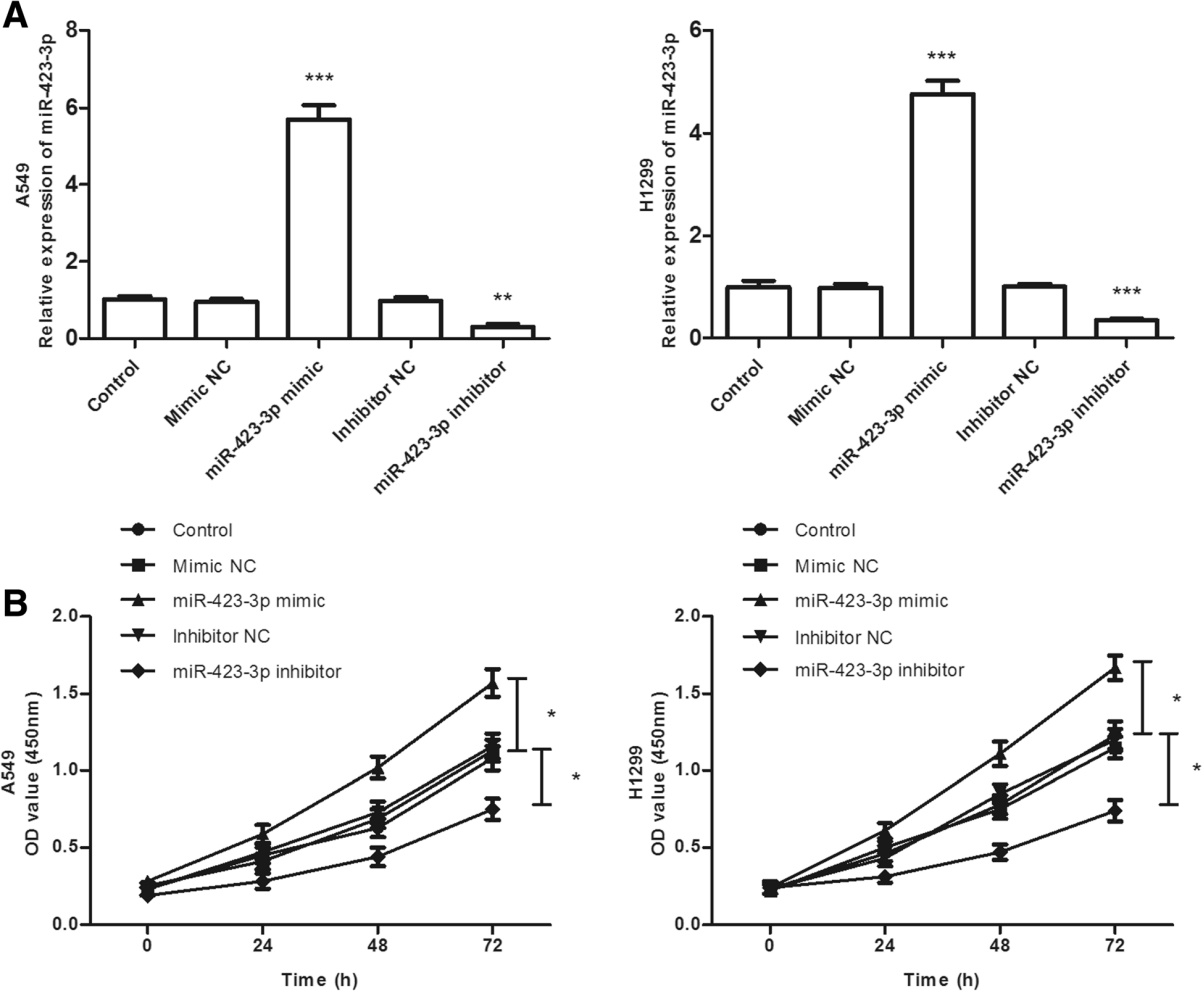
Overexpression of miR-423-3p promotes cell proliferation, while inhibition of miR-423-3p suppresses cell proliferation of A549 and H1299 cells. **a** Expression of miR-423-3p in mimic NC, miR-423-3p mimic, inhibitor NC, or miR-423-3p inhibitor-transfected cells. (**P < 0.01, ***P < 0.001). **b** Effects of miR-423-3p on lung cancer cell proliferation in A549 and H1299 cells. (*P < 0.05)

**Fig. 4:**
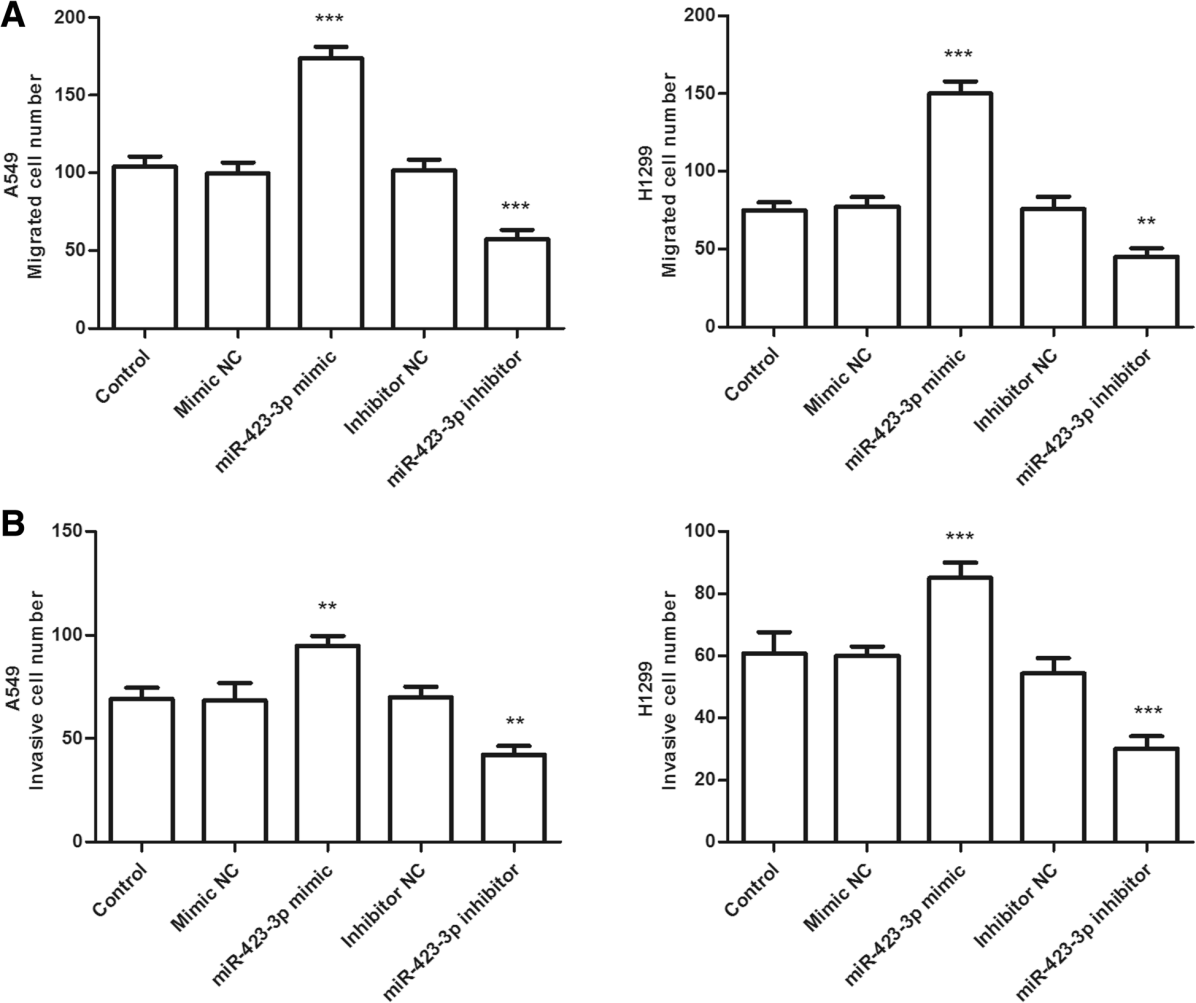
Overexpression of miR-423-3p promotes cell migration and invasion, while inhibition of miR-423-3p suppresses cell migration and invasion in A549 and H1299 cells. **a** Effects of miR-423-3p on lung cancer cell migration. (**P < 0.01, ***P < 0.001). **b** Effects of miR-423-3p on lung cancer invasion. (**P < 0.01, ***P < 0.001)

To further explore the functional role of miR-423-3p in lung cancer, miR-423-3p mimic or miR-423-3p inhibitor was transfected into A549 and H1299 cells. As displayed in Fig. 3a, miR-423-3p expression was significantly increased by miR-423-3p mimics and decreased by miR-423-3p inhibitors in A549 and H1299 cells (all *P* < 0.01). Then we used the CCK-8 assay to determine the cell proliferation capacity. The results showed that the overexpression of miR-423-3p by miR-423-3p mimic promoted cell proliferation, while inhibition of miR-423-3p suppressed cell proliferation (*P* < 0.05, Fig. 3b). Additionally, Transwell migration and invasion assays results indicated that overexpression of miR-423-3p caused a promotion of the migratory and invasive capacities, whereas downregulation of miR-423-3p caused an inhibition of the migratory and invasive capacities of A549 and H1299 cells (all *P* < 0.01,Fig. **4**a and b).

## Discussion

In this study, miR-423-3p was upregulated in lung cancer tissues compared with that in adjacent normal tissues, and in lung cancer cells compared with normal lung epithelial BEAS-2B cell. Overexpression of miR-423-3p in lung cancer tissues was identified to be significantly associated with lymph node metastasis, TNM stage, and poor prognosis of lung cancer patients. Furthermore, miR-423-3p was revealed to function as an oncogene in lung cancer by promoting cell proliferation, migration, and invasion. Taken together, the present results suggest that miR-423-3p functions as an oncogene in lung cancer and may potentially be utilized as a prognostic indicator.

Although substantive progress has been made in diagnosis and targeted therapy for lung cancer, the 5-year overall survival rate of lung cancer is still fairly low [19, 20]. Searching for effective biomarkers for early diagnosis, prognosis, and therapeutic targets for the treatment of lung cancer is still essential. Increasing studies have identified that molecular markers including miRNAs play an important role in the diagnosis and/or prognosis in various cancers [21–24]. For instance, miR-23a as one of the most extensively studied miRNAs in various cancers plays an essential role in the application of cancer diagnosis, prognosis, and treatment [23]. A study indicated that miR-145 was significantly downregulated in hepatocellular carcinoma tissues and had prognostic value in hepatocellular carcinoma [24]. These studies demonstrated that miRNAs have clinical significance for the treatment of cancers.

Some miRNAs have been identified as an oncogene or suppressor gene and involved in the progression of lung cancer [25]. For instance, miR-1269a acts as an onco-miRNA in non-small cell lung cancer (NSCLC) and promotes cancer cell growth by downregulating the expression of SOX6 [26]. miR-140-3p was indicated as a tumor suppressor and had the prognostic and therapeutic role in squamous cell lung cancer [27]. The expression of miR-218 was found significantly downregulated in lung cancer tissues and it acted as a tumor suppressor in lung cancer by targeting IL-6/STAT3 and negatively correlated with poor prognosis [28]. miR-423-3p, located on chromosome 17q11, was reported as an oncogene in several types of cancers, such as breast cancer, head and neck squamous cell carcinoma, and endometrial cancer [29–31]. In the present study, we examined the expression of miR-423-3p and explored its potential role in lung cancer. The results suggested that miR-423-3p was upregulated in lung cancer tissues and cell lines. Moreover, we found miR-423-3p was associated with lymph node metastasis and TNM stage. These results suggested that miR-423-3p may be an oncogene and is involved in the development and progression of lung cancer, which is consistent with other previous studies.

To explore the clinical prognostic significance of miR-423-3p in lung cancer, we performed Kaplan-Meier curve and Cox regression analyses according to the clinical features and overall survival information of lung cancer patients. The results indicated that lung cancer patients with tumors expressing high profiles of miR-423-3p exhibit poorer survival outcome. And miR-423-3p is an independent prognostic factor for overall survival of lung cancer patients. These results indicated that miR-423-3p is an independent prognostic biomarker for lung cancer.

Previous studies also demonstrated that miR-423-3p is closely associated with tumor progression of several types of cancers [32–35]. For instance, in gastric cancer, miR-423-3p is upregulated in gastric cancer tissues and promotes cell proliferation, migration, and invasion in cell lines and animal models [32]. To explore the biological function of miR-423-3p in the progression of lung cancer, we investigated the effects of miR-423-3p on cell proliferation, migration, and invasion of lung cancer cell lines. We found that overexpression of miR-423-3p significantly promoted lung cancer cell proliferation, migration, and invasion, while inhibition of miR-423-3p suppressed cell proliferation, migration, and invasion, compared with controls. In addition, a study by Li et al. showed miR-423-3p is significantly upregulated in colorectal cancer tissues and enhances cell growth through inhibition of p21Cip1/Waf1 in colorectal cancer [33]. Another study by Guan et al. demonstrated miR-423-3p plays an important oncogenic role in laryngeal carcinoma progression via modulation of AdipoR2 [34]. A recent study showed that overexpression of AdipoR1 and AdipoR2 inhibited migration and invasion in NSCLC [36]. In our current study, we observed that miR-423-3p is involved in the development and progression of lung cancer. Therefore, We speculate miR-423-3p may also function as an oncogene role in lung cancer progression through regulating expression of AdipoR2. The detailed molecular mechanisms of miR-423-3p in lung cancer will be investigated in further investigation.

Taken together, this study indicated miR-423-3p is upregulated in lung cancer and promotes cell proliferation, migration, and invasion. Our data suggested that miR-423-3p functions as an oncogene and may act as a prognostic biomarker and therapeutic target for lung cancer.

## Data Availability

All data generated or analyzed during this study are included in this published article.

## Abbreviations

ANOVA: Analysis of Variance
FBS: Fetal bovine serum
NC: Negative control
NSCLC: Non-small cell lung cancer
qRT-PCR: Reverse transcription-quantitative polymerase chain reaction
SD: Standard deviation
TNM: Tumor, node, and metastasis
UTR: Untranslated Region
